# Fostering academic engagement through soft skills and positive emotions: a sustainable development perspective on university education

**DOI:** 10.3389/fpsyg.2025.1622327

**Published:** 2025-07-02

**Authors:** Xiaoling Wang, Xiang Deng, Wan Marzuki Wan Jaafar, Rose Manisah Sulong, Zaida Nor binti Zainudin, Wan Norhayati Wan Othman

**Affiliations:** ^1^Faculty of Educational Studies, Universiti Putra Malaysia, Serdang, Malaysia; ^2^Faculty of Education and Psychological Science, Sichuan University of Science and Engineering, Zigong, Sichuan, China

**Keywords:** soft skills, positive emotions, academic engagement, undergraduate students, education

## Abstract

**Introduction:**

Soft skills, including curiosity, initiative, perseverance, social awareness, adaptability, and leadership, are increasingly recognized as essential for fostering positive emotions and academic engagement in higher education. However, the pathways linking these skills to academic engagement remain underexplored, especially within the Chinese university context.

**Methods:**

This study investigated the relationships among soft skills, positive emotions, and academic engagement among 335 undergraduate students (197 females, 58.8%) from four universities in China, across faculties of Education, Literature, and Management. Standardized questionnaires assessing soft skills, positive emotions, and academic engagement dimensions (absorption, dedication, and vigor) were administered. Data were analyzed using SPSS 26 and AMOS 24. Reliability was confirmed through Cronbach's alpha (≥0.70), and construct validity was evaluated via confirmatory factor analysis (CFA).

**Results:**

CFA indicated an acceptable to excellent fit for both individual scales and the overall measurement model (χ^2^/df = 1.887, RMSEA = 0.052, CFI = 0.928). Structural equation modeling (SEM) results supported hypothesized relationships, demonstrating that soft skills directly predicted academic engagement and indirectly predicted it through positive emotions. Positive emotions significantly mediated the relationship between soft skills and all dimensions of academic engagement (absorption, dedication, vigor).

**Discussion:**

These findings underscore the importance of developing soft skills and fostering positive emotional experiences to enhance academic engagement. Aligning with sustainable development-oriented educational reforms, the results suggest that comprehensive educational approaches promoting soft skills and emotional well-being can effectively support holistic student growth and sustainable academic success.

## 1 Introduction

The transition to higher education represents a critical developmental phase characterized by significant academic and emotional challenges for students. During this period, the most important challenge is student academic engagement. Academic engagement is defined as active participation in learning activities with vigor, dedication, and absorption, is widely recognized as a key predictor of student success and institutional quality (Schaufeli et al., 2002). From a sustainable development perspective, engagement transcends mere academic performance; it is a reflection of holistic student development, encompassing emotional resilience, intellectual curiosity, and a sense of purpose.

Research underscores that engaged students are more likely to exhibit persistence, proactive learning behaviors, and adaptability—traits that not only enhance academic outcomes but also contribute to their broader personal and social development (Krause and Coates, 2008; Kuh, 2009). Conversely, disengagement often leads to diminished emotional and cognitive investment, undermining the potential for students to flourish both academically and personally (Jinghuan et al., 2014; Ross et al., 2011). Thus, enhancing engagement is not only essential for individual achievement but also serves as a proxy for educational quality, reflecting its role in fostering personal growth and institutional excellence (Kuh, 2009; Trowler and Trowler, 2010).

In Chinese higher education, improving student engagement has been identified as a strategic priority for advancing educational quality. While previous research has emphasized the role of cognitive abilities in fostering engagement, growing evidence highlights the equal importance of non-cognitive factors, including emotional states and personality traits (Wang et al., 2025). These factors resonate with sustainable development principles by acknowledging the interconnectedness of personalities and emotional dimensions in shaping students' experiences.

A sustainable development framework emphasizes the need for educational interventions that nurture both the academic and emotional wellbeing of students. By fostering positive emotions, developing personalities, institutions can create supportive environments where students are empowered to pursue their academic and personal goals. Moreover, understanding the mechanisms underlying engagement provides actionable insights for curriculum design, student support systems, and broader institutional reforms.

Beyond individual achievement, academic engagement serves as a vehicle for broader societal contributions, aligning with institutional goals such as regional economic development and global competitiveness (Brint et al., 2008). By embracing a sustainable development approach, educators and policymakers can advance comprehensive reforms that not only improve academic performance but also cultivate the emotional and social capacities necessary for students to thrive in an increasingly complex world.

### 1.1 Soft skills

Academic engagement, a critical predictor of student success, cannot be fully understood without accounting for the influence of individual attributes. Among these, soft skills—non-technical personal qualities such as adaptability, perseverance, curiosity, and social awareness—have emerged as essential determinants of students' academic and emotional wellbeing (Park et al., 2004; Robles, 2012). These skills are instrumental in helping students manage academic demands, foster effective interactions with peers, and achieve personal goals, thereby contributing to sustained engagement in learning activities (Dametto and Noronha, 2019; Zhang et al., 2021).

Soft skills not only enhance students' motivation, self-regulation, and problem-solving abilities but also build emotional resilience, which is crucial for overcoming challenges and maintaining academic focus (Feraco et al., 2022; Waters et al., 2019). Attributes such as adaptability and perseverance enable students to navigate the uncertainties of academic life, while curiosity and social awareness promote collaborative learning and intellectual growth. Consequently, the integration of soft skills into educational frameworks is vital for fostering both individual and institutional success.

Despite their recognized importance in academic and professional contexts, the mechanisms through which soft skills influence academic engagement remain underexplored, particularly in Chinese higher education. Existing research has primarily focused on cognitive determinants of engagement, leaving a significant gap in understanding how non-cognitive attributes, such as emotional and interpersonal competencies, shape students' learning experiences and outcomes (Zhang et al., 2021; Hulme et al., 2013).

Given the strategic priority of enhancing student engagement in China, investigating the role of soft skills offers valuable insights for educational reform. Understanding these mechanisms can inform the design of interventions that not only improve academic engagement but also equip students with competencies essential for long-term success in both academic and professional settings.

### 1.2 Positive emotions

Positive emotions, as conceptualized within the Broaden-and-Build Theory (B&B theory), are integral to enhancing students' cognitive capacities, adaptive behaviors, and overall wellbeing (Fredrickson, 2001). Emotions such as joy, hope, and pride not only alleviate the negative effects of academic stress but also foster intrinsic motivation, goal-setting, and self-regulation, contributing to sustained academic engagement and improved learning outcomes (Huang, 2011; Pekrun et al., 2011). These emotions broaden students' thought-action repertoires, encouraging innovative thinking and the development of resources that support academic success (Jacob et al., 2019; Putwain et al., 2020).

Research underscores the pivotal role of positive emotions in promoting academic engagement. For instance, experiencing enjoyable challenges can inspire persistence, creativity, and deeper learning, while reducing the emotional toll of academic and social challenges (Malykhin et al., 2021; Pekrun, 2006). Moreover, these emotions contribute to students' personal growth, fostering resilience and adaptability during the transition to university life.

Despite extensive evidence linking positive emotions to academic success, limited attention has been given to their interaction with soft skills and the collective influence of these factors on academic engagement. According to the Engine of Wellbeing, soft skills, such as, adaptability and social awareness, can enhance the frequency and intensity of positive emotional experiences, creating a synergistic effect that amplifies students' academic participation (Jayawickreme et al., 2012). Exploring this interplay can provide valuable insights into designing interventions that simultaneously foster emotional wellbeing and academic achievement.

Framed within the principles of sustainable development in education—which emphasizes long-term, holistic student growth—this study contributes to a more comprehensive understanding of student engagement. In the context of Chinese higher education, where improving academic engagement is a pressing concern, examining how soft skills and positive emotions jointly contribute to learning outcomes holds practical significance. By incorporating these findings into institutional strategies, universities can align with sustainability-oriented educational reforms, fostering learners who are not only academically competent but also emotionally resilient and socially responsible.

### 1.3 Present study

Building on these insights, the present study examines the relationships among soft skills, positive emotions, and academic engagement within the context of higher education. Specifically, this study seeks to: (1) evaluate the direct roles of soft skills on positive emotions and academic engagement, and (2) investigate the mediating role of positive emotions in the relationship between soft skills and academic engagement.

To address these objectives, this study employs structural equation modeling (SEM) and mediation analysis, offering a comprehensive understanding of the mechanisms through which soft skills influence students' emotional and academic outcomes.

**Figure 1:**
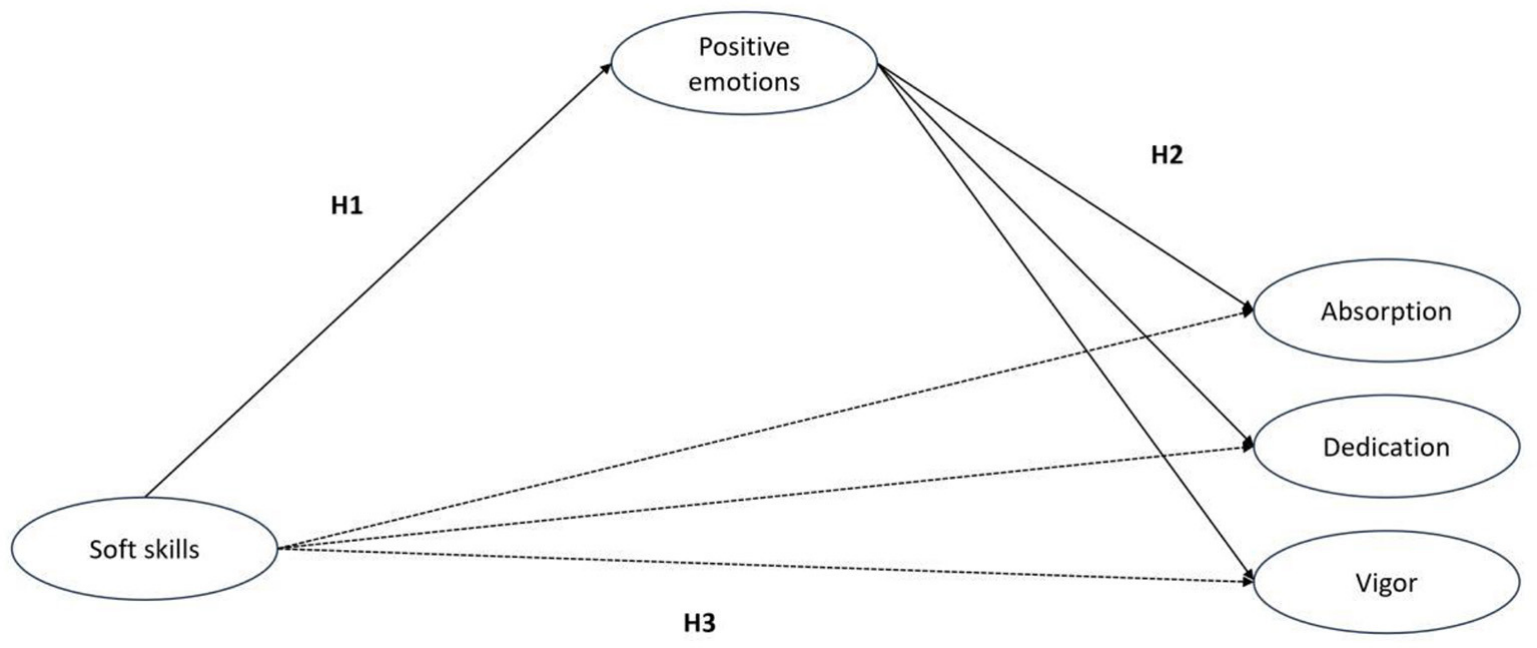
The hypothesized model.

The conceptual framework (see Figure 1) is grounded in existing theories and empirical findings, leading to the following hypotheses:

H1: Soft skills positively associated with positive emotions.

H2: positive emotions positively associated with academic engagement (H2a-absorption, H2b-dedication and H2c-vigor)

H3: Soft skills positively associated with academic engagement (H3a-absorption, H3b-dedication and H3c-vigor).

H4: Positive emotions mediate the relationship between soft skills and academic engagement (H4a-absorption, H4b-dedication and H4c-vigor).

## 2 Methods

### 2.1 Participants

Total of 335 participants enrolled in this study, including 197 (58.8%) female student. The participants were drawn from four universities, three faculty. The specific demographic characteristics of the sample are summarized in Table 1.

**Table 1:** Demographic statistics (*N* = 335).

**Demographic information**	**Frequency (*n*)**	**Percent (%)**
**Faculty**		
Education	122	36.40%
Literature	103	30.70%
Management	110	32.80%
**Gender**		
Male	138	41.20%
Female	197	58.80%
**Grade**		
Freshman	130	38.80%
Sophomore	59	17.60%
Junior	119	35.50%
Senior	27	8.10%
**Total**	335	100%

### 2.2 Materials

The reliability and validity of the scales used in this study are summarized in Table 2. All constructs achieved Cronbach's alpha values higher than 0.7, indicating satisfactory internal consistency (Guilford, 1950). As the study was conducted in China, the original English scales were translated into Chinese with permission from the original authors. A rigorous back-translation procedure was employed by two bilingual experts to ensure linguistic and conceptual equivalence.

**Table 2:** Construct items of measurement model.

**Constructs**	**Factor loading**	**AVE**	**CR**	**Cronbach's alpha**
**Soft skills**				
**Curiosity**		0.608	0.885	0.888
SSC1	0.697			
SSC2	0.690			
SSC3	0.824			
SSC4	0.846			
SSC5	0.828			
**Initiative**		0.417	0.738	0.746
SSI1	0.664			
SSI4	0.518			
SSI5	0.638			
SSI6	0.742			
**Perseverance**		0.521	0.844	0.845
SSP2	0.732			
SSP3	0.657			
SSP4	0.732			
SSP5	0.770			
SSP6	0.712			
**Social awareness**		0.534	0.771	0.762
SS2	0.843			
SS3	0.724			
SS5	0.605			
**Adaptability**		0.535	0.822	0.828
SSA1	0.701			
SSA2	0.771			
SSA3	0.725			
SSA6	0.728			
**Leadership**		0.557	0.832	0.846
SSL1	0.824			
SSL2	0.765			
SSL3	0.776			
SSL4	0.601			
**Positive emotions**		0.562	0.920	0.926
PE1	0.717			
PE2	0.685			
PE3	0.711			
PE4	0.733			
PE5	0.757			
PE7	0.740			
PE8	0.772			
PE9	0.801			
PE10	0.821			
**Academic engagement**				
**Absorption**		0.768	0.908	0.908
AEA1	0.852			
AEA2	0.891			
AEA3	0.885			
**Dedication**		0.780	0.914	0.914
AED1	0.879			
AED2	0.913			
AED3	0.857			
**Vigor**		0.684	0.861	0.843
AEV1	0.934			
AEV2	0.923			
AEV3	0.572			

SSC, Soft skills-curiosity; SSI, Soft skills-initiative; SSP, Soft skills-perseverance; SSS, Soft skills-social awareness; SSA, Soft skills-adaptability; SSL, Soft skills-leadership; PE, Positive emotions; AEA, Academic engagement-Absorption; AEV, Academic engagement-Vigor; AED, Academic engagement-Dedication.

#### 2.2.1 The soft skills scale

The soft skills scale developed by Feraco et al. (2022) was employed, comprising six subscales: curiosity, initiative, perseverance, social awareness, adaptability, and leadership. Each subscale contains six items, except for the leadership subscale, which includes four items. Responses were recorded on a 6-point Likert scale (1 = strongly disagree, 6 = strongly agree), and average scores were calculated.

**Curiosity:** CFA revealed one item with a factor loading < 0.50, which was subsequently removed. The revised model, with five items, demonstrated good fit indices: χ^2^/*df* = 3.071, *p* = 0.009, GFI = 0.981, AGFI = 0.943, CFI = 0.989, RMSEA = 0.079. Factor loadings ranged from 0.690 to 0.846.

**Initiative:** Following the removal of one low-loading item, the revised model with five items yielded acceptable factor loadings (0.518–0.742) and good fit indices: χ^2^/*df* = 1.690, *p* = 0.185, GFI = 0.995, CFI = 0.995, RMSEA = 0.045.

**Perseverance:** One item was removed due to a factor loading < 0.50. The revised model, with five items, acceptable factor loadings (0.657 to 0.770), achieved satisfactory fit: χ^2^/*df* = 2.584, *p* = 0.024, GFI = 0.985, CFI = 0.987, RMSEA = 0.069.

**Social awareness:** Three items were excluded. The final model, with three items, was just-identified with a perfect fit. Factor loadings ranged from 0.605 to 0.843.

**Adaptability:** Two low-loading items were removed. The revised model, with four items, acceptable factor loadings (0.701 to 0.771), showed excellent fit: χ^2^/*df* = 1.397, *p* = 0.247, GFI = 0.996, CFI = 0.990, RMSEA = 0.034.

**Leadership:** All four items demonstrated acceptable loadings (0.601–0.824). The model achieved excellent fit: χ^2^/*df* = 0.709, *p* = 0.370, GFI = 0.999, CFI = 1.000, RMSEA = 0.000.

#### 2.2.2 The positive emotions scale

The Positive and Negative Affect Scale (PANAS; Watson et al., 1988) was used to measure positive emotions. This subscale consists of 10 items rated on a 5-point Likert scale (1 = not at all, 5 = extremely). One item with a factor loading < 0.50 was removed. The revised model, with nine items, exhibited good fit: χ^2^/*df* = 3.149, *p* < 0.001, GFI = 0.940, CFI = 0.968, RMSEA = 0.080. Factor loadings ranged from 0.723 to 0.803.

#### 2.2.3 Academic engagement scale

The academic engagement scale, developed by Schaufeli and Bakker (2003), consists of nine items measuring absorption, dedication, and vigor. Responses were recorded on a 7-point Likert scale (0 = almost never to 6 = always), and average scores were calculated. CFA confirmed the construct validity of the scale, with excellent fit indices: χ^2^/*df* = 1.183, *p* = 0.224, GFI = 0.983, CFI = 0.998, RMSEA = 0.023.

#### 2.2.4 Reliability and validity test

The reliability and validity of the constructs were assessed using several statistical indicators, including Cronbach's alpha, composite reliability (CR), average variance extracted (AVE), and factor loadings (see Table 2).

##### 2.2.4.1 Reliability

Reliability reflects the internal consistency of a construct, ensuring that the items within a scale measure the same underlying concept. In this study, all constructs demonstrated acceptable reliability, as evidenced by Cronbach's alpha (ranging from 0.746 to 0.926) and CR (ranging from 0.738 to 0.920) values above the recommended threshold (Table 2).

##### 2.2.4.2 Validity

Validity ensures that a scale accurately measures the intended construct. Convergent validity was assessed using factor loadings and AVE. Factor loadings above 0.50 and AVE values above 0.50 are considered indicative of convergent validity (Fornell and Larcker, 1981). Most constructs had acceptable validity, AVE value of initiative is < 0.50, but the CR of initiative is above than 0.7, thus, the convergent validity of initiative is acceptable.

### 2.3 Model fit

To evaluate the adequacy of the measurement and structural models, both first-order and second-order confirmatory factor analyses (CFA) were conducted for the constructs of positive emotions, academic engagement, and soft skills. The results of the CFA demonstrated varying levels of model fit, as summarized in Table 3.

**Table 3:** Summary of value goodness-of-fit-index model.

**Goodness of fit index**	**Values results**			
	**Second-order CFA soft skills**	**First-order CFA positive emotions**	**First-order CFA academic engagement**	**Measurement model**
CMIN/DF	2.949	3.149	1.183	1.887
RESEA	0.076	0.08	0.023	0.052
CFI	0.905	0.968	0.998	0.928
TLI	0.893	0.957	0.997	0.922
IFI	0.905	0.968	0.998	0.928
GFI	0.828	0.94	0.983	0.809

For the measurement model, the final indices indicated an acceptable fit (χ^2^/*df* = 1.887, GFI = 0.809, CFI = 0.928, TLI = 0.922, RMSEA = 0.052), consistent with the thresholds suggested by Hair et al. (2010).

The structural model similarly demonstrated an acceptable overall fit, with indices as follows: χ^2^/*df* = 2.191, GFI = 0.784, CFI = 0.903, IFI = 0.904, TLI = 0.896, and RMSEA = 0.06.

### 2.4 Data collection

Prior to the commencement of the study, ethical approval was obtained. To uphold participants' rights and autonomy, an electronic informed consent form was administered through the online survey platform. Participants were provided with detailed information about the study's purpose, assurances of anonymity and confidentiality, and their right to withdraw at any stage without penalty. Consent was indicated by selecting “Agree” before proceeding to the survey.

Data collection was conducted via an online survey platform using a translated version of the standardized instrument. The translation process adhered to Brislin's (1983) guidelines to ensure linguistic accuracy and cultural equivalence. The survey comprised sections on demographic information, soft skills, positive emotions, and academic engagement and required approximately 10–15 min to complete. Automated platform mechanisms ensured participant anonymity throughout the process.

### 2.5 Data analysis

Descriptive, reliability, and structural analyses were conducted using SPSS version 26 for descriptive and inferential statistics, and AMOS version 24 for structural equation modeling (SEM). The analyses were structured to address the study's objectives and test the associated hypotheses systematically.

## 3 Results

### 3.1 Descriptive statistics and correlations

Table 4 shows the basic information for the variables in the study, including soft skills, positive emotions, and dimensions of academic engagement (absorption, dedication, and Vigor).

**Table 4:** Means, standard deviations and correlations of variables.

**Variables**	**Mean**	**SD**	**1**	**2**	**3**	**4**
Soft skills	4.41	0.59				
Positive emotions	3.40	0.61	0.630^**^			
Absorption	5.27	0.87	0.535^**^	0.486^**^		
Dedication	5.32	0.94	0.559^**^	0.542^**^	0.787^**^	
Vigor	5.03	1.02	0.524^**^	0.562^**^	0.584^**^	0.642^**^

^*^p < 0.05,

** *p* < 0.01.

### 3.2 Path analysis results

The SEM analysis provided robust evidence supporting the hypothesized relationships among soft skills, positive emotions, and the dimensions of academic engagement (absorption, dedication, and Vigor). The standardized path coefficients, *p*-values, and confidence intervals confirm both the direct and indirect effects (Table 5).

**Table 5:** Path analysis results.

**Relation**	**SDT Estimate**	***P***	**95% Confidence Interval**	
			**Lower**	**Upper**
**Direct relation**				
H1: SS → PE	0.672	0.000	0.595	0.741
H2a: PE → AEA	0.349	0.000	0.183	0.539
H2b: PE → SED	0.426	0.000	0.267	0.601
H2c: PE → SEV	0.498	0.000	0.333	0.660
H3a: SS → SEA	0.361	0.001	0.168	0.528
H3b: SS → SED	0.335	0.001	0.154	0.491
H3c: SS → SEV	0.251	0.003	0.098	0.416
**Indirect relation**				
H4a: SS → → SEA	0.235	0.000	0.124	0.384
H4b: SS → → SED	0.286	0.000	0.180	0.432
H4c: SS → → SEV	0.335	0.000	0.224	0.465

Soft skills showed a positive association with positive emotions (β = 0.672), supporting H1. This finding underscores the critical role of soft skills in fostering positive emotional states, which align with the Engine of Wellbeing model, emphasizing the psychosocial benefits of soft skills.

Positive emotions were significantly associated with the dimensions of academic engagement, including absorption (β = 0.349), dedication (β = 0.426), and vigor (β = 0.498), supporting H2a-H2c. These results highlight the critical role of positive emotional experiences in enhancing students' academic engagement.

Soft skills were also directly associated with academic engagement, demonstrating positive relationships with absorption (β = 0.361), dedication (β = 0.335), and vigor (β = 0.251), supporting H3a–H3c.

The structural model accounted for 45.1% of the variance in positive emotions and 42.1%, 48.5%, and 48.0% of the variance in absorption, dedication, and Vigor, respectively. The model explained 45–48% of the variance across constructs, indicating robust predictive power.

### 3.3 Mediation effect

The mediation analysis revealed that positive emotions serve as a significant mediator in the relationships between soft skills and academic engagement.

Specifically: soft skills were indirectly associated with absorption via positive emotions (β = 0.235; 95% CI [0.124, 0.384]), supporting H4a. A significant indirect association was also found between soft skills and dedication through positive emotions (β = 0.286; 95% CI [0.180, 0.432]), supporting H4b. The indirect relationship between soft skills and vigor, mediated by positive emotions, was also significant (β = 0.335; 95% CI [0.224, 0.465]), supporting H4c.

**Figure 2:**
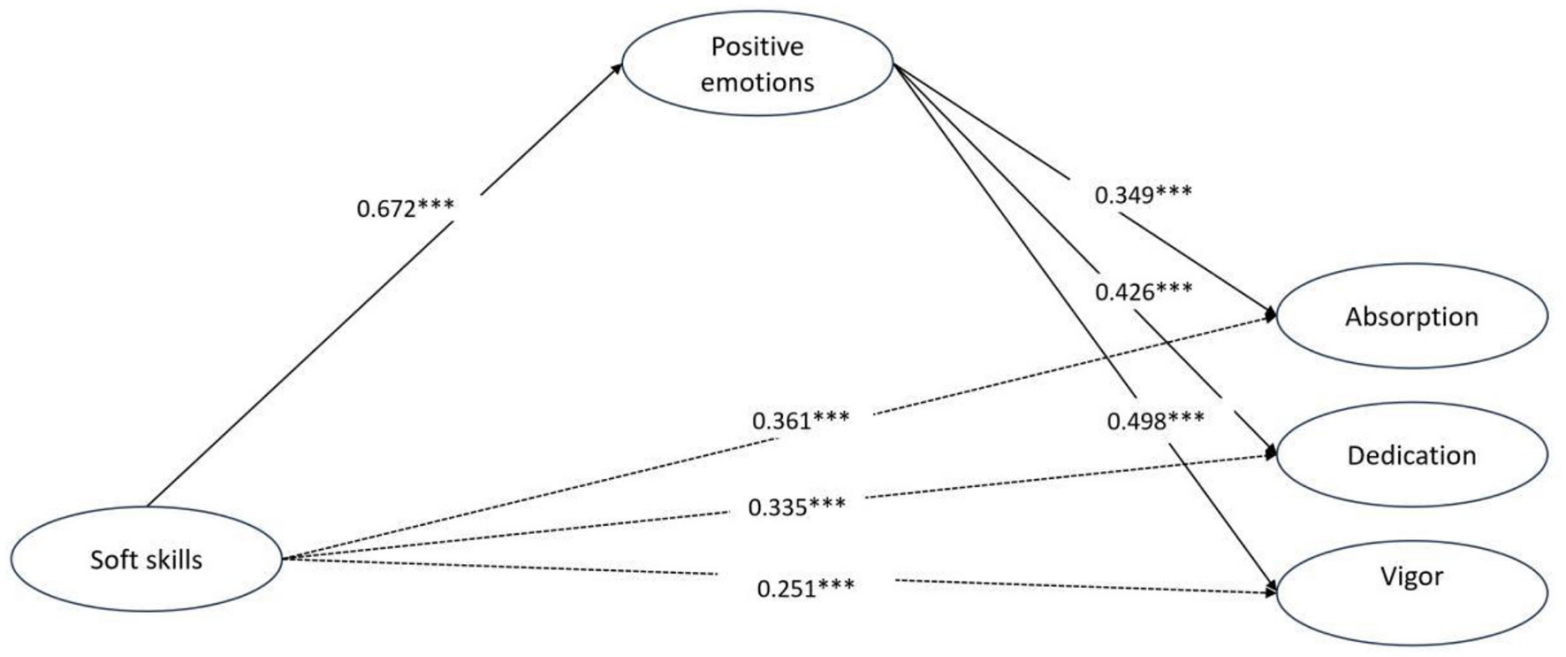
Path model and construct estimates. ^***^p < 0.001. *N* = 335. Coefficients were standardized.

These findings emphasize the dual role of soft skills in directly fostering academic engagement and indirectly enhancing it through positive emotions. The results align with theories highlighting the interplay between personal competencies, emotional wellbeing, and academic outcomes (see Figure 2).

## 4 Discussion

This study provides significant insights into the relationships among soft skills, positive emotions, and academic engagement, highlighting both direct and indirect pathways. By employing SEM and mediation analysis, the results contribute to a nuanced understanding of these constructs within context of student engagement.

The results confirm that soft skills positively predict positive emotions, supporting previous findings that students with strong soft skills are better equipped to navigate emotional and academic challenges. This is consistent with Feraco et al. (2022), who emphasize the role of soft skills in stress management and emotional regulation, and Dametto and Noronha (2019), who highlight attributes such as curiosity, perseverance, and social awareness as critical contributors to positive emotional experiences. These findings suggest that students with robust soft skills may possess greater emotional resilience, enabling them to respond more effectively to the demands of academic environments.

Additionally, the study demonstrates significant relationship between soft skills and academic engagement, reinforcing the notion that these non-cognitive personalities are critical drivers of students' academic involvement. This finding is consistent with Aryani et al. (2021), who argued that soft skills enhance psychological capital, enabling individuals to sustain engagement across contexts—from high school to university and eventually the workplace. Besides, Feraco et al. (2022) emphasized that soft skills foster self-regulated learning and learning motivation, both of which are essential for meaningful academic achievement. Furthermore, it has been suggested that soft skills show significant associations with students' academic performance (Keng, 2024). These insights suggest that soft skills not only facilitate the development of positive emotions but also strengthen students' ability to remain engaged in academic tasks.

The mediation of positive emotions between soft skills and academic engagement further validates the B&B Theory (Fredrickson, 2001), which suggests that positive emotions broaden cognitive resources and foster adaptive behaviors. Students with high levels of soft skills tend to experience positive emotions, which in turn enhance and engagement. This finding is consistent with previous research, which identify positive emotions as a critical factor in sustaining academic involvement (Oriol-Granado et al., 2017). Moreover, the results complement the Engine of Wellbeing Model, which underscores the interplay between personal traits and emotional processes in academic success (Jayawickreme et al., 2012). By highlighting the mediation of positive emotions, it contributes to deeper understandings of the mechanisms through which soft skills impact student engagement.

This study provides critical theoretical contributions by illuminating the integral role of soft skills in shaping positive emotions and academic engagement, thereby enriching the understanding of student engagement through established psychological frameworks. Firstly, the findings affirm the foundational tenets of the Engine of Wellbeing Model, which underscores the importance of personal competencies such as curiosity, adaptability, and perseverance in promoting emotional wellbeing and fostering adaptive behaviors. By demonstrating the direct and indirect roles of soft skills on academic engagement, this research extends the applicability of the model to educational settings, offering a nuanced perspective on how these competencies serve as psychological resources that enhance students' emotional and academic experiences.

Secondly, the study substantiates the B&B Theory of positive emotions by demonstrating how positive emotional states contribute to higher levels of engagement across its dimensions—absorption, dedication, and Vigor. The findings suggest that positive emotions function as critical mechanisms by which soft skills amplify academic engagement, reinforcing the theory's assertion that these emotional states enhance adaptive behavior and cognitive engagement (Saleem et al., 2022). By integrating these theories, the study offers a comprehensive framework that links personal traits, emotional processes, and academic outcomes, thereby addressing gaps in the literature and advancing theoretical discourse in the domains of educational psychology and student wellbeing.

Besides, from a practical perspective, the study's findings highlight actionable strategies aligned with the sustainable development-oriented educational framework, clarifying its role by emphasizing holistic student growth. Specifically, the dual influence of soft skills—directly enhancing academic engagement and indirectly facilitating this relationship through positive emotions—provides clear guidance for educators, curriculum designers, and policymakers. To pragmatically leverage these insights, educators and curriculum developers should explicitly incorporate structured and measurable initiatives aimed at enhancing core soft skills such as curiosity, adaptability, and perseverance. Practical approaches include embedding experiential learning activities, scenario-based exercises, and collaborative projects within curricula, which directly cultivate these competencies in alignment with broader sustainability objectives. Additionally, educational stakeholders—such as policymakers, administrators, and student support services—are encouraged to implement and support evidence-based emotional wellness programs. Practices like mindfulness training, positive psychology interventions, and structured emotion-regulation workshops can systematically improve students' positive emotional experiences, thereby strengthening both their academic engagement and emotional resilience. Such strategies offer educators and institutions concrete pathways for fostering comprehensive student development, directly linking educational practices to long-term sustainable educational outcomes.

Despite its contributions, this study has limitations. First, the cross-sectional design precludes causal inferences, highlighting the need for longitudinal research to capture the dynamic interactions among soft skills, positive emotions, and academic engagement over time. Second, the study's sample was drawn from a single educational context, limiting the generalizability of the findings. Future studies should explore these relationships in diverse cultural and institutional settings. Finally, while this study focused on positive emotions as a mediator, other potential mediators—such as resilience, self-efficacy, or social support—merit further examination to provide more comprehensive understandings of the pathways influencing academic engagement.

## 5 Conclusion

In conclusion, this study clarifies and extends the sustainable development-oriented educational framework by emphasizing the integral role of soft skills and positive emotions in fostering holistic student engagement. By identifying both direct and mediated effects, the findings provide valuable theoretical grounding and actionable practical implications for stakeholders. Specifically, educational institutions and policymakers are encouraged to adopt integrated curricular and co-curricular strategies explicitly designed to cultivate essential soft skills and enhance positive emotional experiences. Such comprehensive educational initiatives align closely with sustainable development goals by promoting long-term academic success, emotional resilience, and overall student wellbeing.

## Data Availability

The original contributions presented in the study are included in the article/supplementary material, further inquiries can be directed to the corresponding author.
